# Seroepidemiology of *Toxocara Canis* infection among primary schoolchildren in the capital area of the Republic of the Marshall Islands

**DOI:** 10.1186/1471-2334-14-261

**Published:** 2014-05-15

**Authors:** Chung-Jung Fu, Ting-Wu Chuang, Huei-Shan Lin, Chih-Hsiung Wu, Yung-Ching Liu, Mailynn K Langinlur, Min-Yun Lu, Wesley Wei-Wen Hsiao, Chia-Kwung Fan

**Affiliations:** 1 Graduate Institute of Biomedical Electronics and Bioinformatics, National Taiwan University, Taipei, Taiwan; 2 Department of Surgery, Shuang Ho Hospital, Taipei Medical University, New Taipei City, Taiwan; 3 College of Medicine, Taipei Medical University, Taipei, Taiwan; 4 Center for International Tropical Medicine, College of Medicine, Taipei Medical University, Taipei, Taiwan; 5 Superintendent Office, Shuang Ho Hospital, Taipei Medical University, New Taipei City, Taiwan; 6 Section of Infectious Diseases, Taipei Medical University Shuang Ho Hospital, Taipei, Taiwan; 7 Department of Internal Medicine, School of Medicine, College of Medicine, Taipei Medical University, Taipei, Taiwan; 8 Department of Public Health, Ministry of Health, The Republic of the Marshall Islands, Taipei, Taiwan; 9 Institute of Atomic and Molecular Sciences, Academia Sinica, Taipei, Taiwan; 10 Master Program in Global Health and Development, College of Public Health and Nutrition, Taipei Medical University, Taipei, Taiwan

**Keywords:** *Toxocara canis* (*T. canis*), Western blotting, Primary school children (PSC), Republic of the marshall islands (RMI)

## Abstract

**Background:**

Toxocariasis, which is predominantly caused by *Toxocara canis* (*T. canis*) infection, is a common zoonotic parasitosis worldwide; however, the status of toxocariasis endemicity in the Republic of the Marshall Islands (RMI) remains unknown.

**Methods:**

A seroepidemiological investigation was conducted among 166 primary school children (PSC) aged 7–12 years from the capital area of the RMI. Western blots based the excretory-secretory antigens of larval *T. canis* (TcES) was employed, and children were considered seropositive if their serum reacted with TcES when diluted at a titer of 1:64. Information regarding demographic characteristics of and environmental risk factors affecting these children was collected using a structured questionnaire. A logistic regression model was applied to conduct a multivariate analysis.

**Results:**

The overall seropositive rate of *T. canis* infection was 86.75% (144/166). In the univariate analysis, PSC who exhibited a history of feeding dogs at home (OR = 5.52, 95% CI = 1.15–26.61, *p* = 0.02) and whose parents were employed as nonskilled workers (OR = 2.86, 95% CI = 1.08–7.60, *p* = 0.03) demonstrated a statistically elevated risk of contracting *T. canis* infections. Cleaning dog huts with gloves might prevent infection, but yielded nonsignificant effects. The multivariate analysis indicated that parental occupation was the critical risk factor in this study because its effect remained significant after adjusting for other variables; by contrast, the effect of dog feeding became nonsignificant because of other potential confounding factors. No associations were observed among gender, age, consuming raw meat or vegetables, drinking unboiled water, cleaning dog huts with gloves, or touching soil.

**Conclusions:**

This is the first serological investigation of *T. canis* infection among PSC in the RMI. The high seroprevalence indicates the commonness of *T. canis* transmission and possible human risk. The fundamental information that the present study provides regarding *T. canis* epidemiology can facilitate developing strategies for disease prevention and control.

## Background

The ascarids that cause human toxocariasis are *Toxocara canis* (*T. canis*) and, likely to a lesser extent, *Toxocara cati* (*T. cati*). The definitive hosts of *T. canis* and *T. cati* are dogs and cats, respectively; these ascarids inhabit the lumen of the small intestine [1]. Worldwide surveys of *T. canis* occurrence have indicated a prevalence ranging from 86% to 100% in pups and 1% to 45% in adult dogs [2,3]. Humans are one of several accidental hosts, and are primarily infected by ingesting parasite eggs or, to a lesser extent, by consuming chicken or cow livers [4].

Although human infections with *Toxocara* spp. are typically asymptomatic, larval migration into the internal organs via the blood can cause various clinical syndromes including visceral larva migrans and ocular larva migrans. The manifestation of symptoms in human toxocariasis depends on multiple factors, including which organs are affected and the magnitude of the infection [3,5].

Young children up to the age of 12 years appear to be the primary population susceptible to *T. canis* infection because of dirt pica, poor hygiene, or frequent contact with dogs [3,6]. Multiple reports have indicated that child toxocariasis is associated with endomyocarditis, generalized lymphadenopathy, endophthalmitis, asthma, hepatosplenomegaly, and meningoencephalitis [7-11]. Considerable interest has been directed toward the role of *T. canis* infection in epilepsy, and particularly in partial epilepsy [12-14].

In humans, parasites cannot mature to the adult stage; thus, examining stool for parasites and eggs is not useful. Making a direct parasitological diagnosis by using biopsy is extremely difficult; thus, serological methods are the diagnostic mainstay. Serological diagnoses of toxocariasis primarily rely on a *T. canis* larval excretory-secretory (TcES) antigen-based enzyme-linked immunosorbent assay (ELISA) of *T. canis*[3,5]. The seroprevalence of *T. canis* infection among children in various countries has been reported to range from 4% to 86% according to TcES-ELISA [15-17]. No reports on the seroprevalence of *T. canis* infection in children in Micronesian areas are available, and its status remains unknown among children who live in the Republic of the Marshall Islands (RMI).

The sensitivity and specificity of TcES-ELISA, when 1:32 was used as the threshold titer for positivity, have recently been estimated at 78% and 92%, respectively [18,19]; however, antigenic cross-reactivity (e.g., with *Ascaris lumbricoides*) reduces the usefulness of such assays, particularly in areas where polyparasitism is common [19]. Western blotting based on the fractionated, native, and excretory-secretory antigens of *T. canis* larvae (TcES-WB) can yield superior specificity levels, exhibiting reactivity to bands of low molecular weights (24–32 kDa) that were proven to be specific to *T. canis* infection [19]. In the present study, TcES-WB was used to detect *T. canis*-specific Immunoglobulin G (IgG) and estimate the seroprevalence of *T. canis* infection among primary schoolchildren (PSC) living in the capital area of Majuro of the RMI.

## Methods

### Geography of the Republic of the Marshall Islands and Majuro Atoll

The RMI is an island nation situated in the central Pacific Ocean between 4° and 14° North latitude and 160° and 173° East longitude. The country comprises approximately 1,225 islands and islets and lies in two parallel chains of 29 low-lying atolls: the Eastern Ratak (Sunrise) and Western Ralik (Sunset) chains of atolls and islands. The RMI is divided into 24 municipalities and Majuro, Ebeye, Wotje, and Jaluit are its major district centers. The Majuro Atoll, a large coral atoll of 64 islands, is a legislative district of the Ratak chain of the Marshall Islands. The Majuro Atoll has a land area of 3.7 mi^2^ and encloses a lagoon of 114 mi^2^. Similar to other atolls in the Marshall Islands, Majuro consists of extremely narrow land masses, on which a person can walk from the lagoon side to the ocean side within minutes. The primary population center, also named Majuro, is the capital of and largest city in the RMI. The RMI has a total population of 52,560. Its characteristic climate is tropical, and a long wet season occurs between June and November. The economy of the RMI primarily relies on agriculture, fishery, and support from the United States. The major ethnic group is Micronesian [20].

### Study population and participant selection

This study was conducted among PSC in Majuro, the capital city of the RMI. Public health nurses collected blood specimen of PSC, after informed consent was obtained from PSC or parents/guardians, from schools located in urban and suburban areas. These well-trained public health nurses interviewed the enrolled schoolchildren by using the structured questionnaire we designed in a previous study [21]. Basic demographic data regarding age, gender, parental occupation, height, weight, self-reported health status, and urbanization levels were collected during the interview. To maintain a sufficient sample size in the analysis, the children were divided into three age groups: Group 1: < 7 years; Group 2: 7–9 years; and Group 3: > 9 years. Parental occupation was classified into two categories and six levels: skilled workers and nonskilled workers. More than 60% of parents were employed at the semiskilled level (e.g., truck drivers, factory workers, or salespeople), which was classified as nonskilled work in the analysis. Among the 166 PSC enrolled in the study, the sex ratio was 1.18 (male to female) and the mean age was 9.5 ± 2.3 years.

Multiple environmental risk factors were investigated in this study. Data regarding contact with dogs, dog feeding behavior, cleaning dog huts while wearing gloves, consuming raw or undercooked meat, and drinking untreated or unboiled water were included in the multivariate analysis.

### Ethical approval

The research protocols were approved by the Institutional Review Board of Shuang Ho Hospital, Taipei Medical University (TMU-JIRB-No. 201306003) and also it was approved by Ministry of Health, RMI.

### *Toxocara* egg culture

Adult *T. canis* were collected from the stools of stray dogs that had been treated with mebendazole (Janssen-Cilag, High Wycombe, UK). Eggs, harvested from the anterior third of the uteri of female worms were cultured according to the method of Fan et al. [21,22]. Briefly, the eggs were stirred in 1% (w/v) sodium hypochlorite solution, left for 5 min at room temperature and then centrifuged (5 min at 2000 × g). After the eggs were washed twice with distilled water and once with 2% formalin, they were resuspended in 2% formalin and placed in a 250-mL Erlenmeyer flask, to which additional 2% formalin was added until attaining a 1-cm-deep layer of liquid. The flask was sealed with ParafilmH (SPI Supplies, West Chester, PA, USA) and left at room temperature for 8–9 weeks, undergoing gentle weekly agitation. The suspended eggs (then containing second-stage larvae) were then stored at 4°C until use.

### Preparation of *T. canis* larval excretory-secretory antigens

An identical protocol has been used in our previous studies [21,22]. When larvae were required, the embryonated eggs were hatched under aseptic conditions; the eggs were washed with sterile phosphate-buffered saline (PBS), resuspended in sterile 1% (w/v) sodium hypochlorite, and incubated in an atmosphere containing 5% CO_2_, for 30 min at 37°C. After several washes in sterile PBS containing three antibiotics (100 IU of penicillin, 250 mg of streptomycin, and 25 mg/mL of nystatin; Biochrom KG, Berlin, Germany), the pelleted larvae were resuspended in RPMI-1640 medium (JRH Biosciences, Lenexa, KS, USA) containing the same concentrations of the three antibiotics. Motile larvae were collected using a modified Baermann apparatus, which was left in an atmosphere containing 5% CO_2_, for 12 h at 37°C. The larvae were transferred to 50-mL tissue culture flasks (BD Biosciences, Franklin Lakes, NJ, USA) containing fresh RPMI-1640 medium and antibiotics, yielding 10^4^ larvae/mL, then incubated in an atmosphere containing 5% CO_2_ at 37°C. The supernatant medium in each culture, which contained TcES antigens, was collected (and replaced with fresh medium) weekly for 3–4 weeks. These samples were pooled, and then centrifuged to precipitate the debris. The resulting supernatant solution was sterilized using filtration through a membrane (exhibiting 0.2-mm pores) and then dialyzed (at a molecular weight cut-off of 6000–8000 kDa) against PBS at 4°C, either for 12 h or until the phenol red in the medium could no longer be observed. The protein content of the dialysate was estimated before the dialysate was concentrated using lyophilization (Labconco, Kansas City, MO, USA) and stored at -70°C until use.

### Western-blot analysis

The same protocol has been used in our previous studies [21,22]. The larval excretory-secretory antigens were used in western blots to test each serum sample for *Toxocara*-specific IgG. Briefly, the TcES (9 mg/slab) were separated using 12.5% sodium-dodecylsulphate polyacrylamide-gel electrophoresis (SDS-PAGE) and then transferred to strips of nitrocellulose membrane (Amersham Biosciences, Little Chalfont, UK) in a semiblotter (Hoffer, Fremont, CA, USA.). The membrane strips were then incubated with sera, each diluted at 1:64, before employing a Western Lightning H kit (PerkinElmer Life Sciences, Boston, MA, USA) to detect immunoreactions. When a serum reacted with any of the low molecular-weight *T. canis*-specific bands (24, 28, 30, or 35 kDa) [19], a child was considered seropositive.

### Statistical analysis

To evaluate the associations between demographic characteristics and *T. canis* infection, a chi-square test was used to compare the proportions of infection based on gender, age group, parental occupation, and urbanization level. A logistic regression model was applied to investigate multiple environmental risk factors associated with *T. canis* infection. All statistical analyses were conducted using SAS Version 9.3 (SAS Institute, Inc., Cary, NC, USA).

## Results

Of the 166 PSC serum samples, 144 (86.75%; 144/166) tested positive for *Toxocara* IgG antibodies according to TcES-WB. The overall seroprevalence was 83.33% (75/90) in boys and 90.79% (69/76) in girls (Table 1). The seroprevalence was highest (95.45%, 21/22) in Group 1 (<7 years), followed by 88.14% (52/59) in Group 3 (7–9 years), and 83.53% (71/85) in Group 3 (>9 years; Table 1). PSC who lived in urban and suburban areas demonstrated seropositive values for the IgG antibody against TcES of 90.32% (84/93) and 82.19% (60/73), respectively (Table 1). The seropositive rates among those whose parents were nonskilled or skilled workers were 89.91% (98/109) and 75.68% (28/37), respectively (Table 1). The risk factors analysis indicated that the seropositive rates were 88.05% (140/159) among PSC who exhibited histories of feeding dogs, 88.89% (56/63) among those who cleaned dog huts, 80.0% (8/10) among those who cleaned dog huts while wearing gloves, 87.74% (136/155) among those who played with soil, 87.41% (125/143) among those who consumed frozen or raw foods, 85.07% (114/134) among those who ate raw vegetables, and 88.72% (118/133) among those who drank untreated or unboiled water (Table 2). In the multivariate logistic regression analysis, gender, age, parental occupation, and risk factors were included in the regression model. As indicated by Table 3, gender was not significantly associated with seropositivity for the *T. canis* antibody when conducting the uni- or multivariate logistic regression analyses (*p* > 0.05). In addition, age was not a critical factor related to *T. canis* infection, because no significant differences in seroprevalence were observed among the age groups when conducting the uni- or multivariate logistic regression analyses (*p* > 0.05).

**Table 1:** **Demographic characteristics of *Toxocara canis *** **infection among primary school children in the capital areas in the Republic of the Marshall Islands**

**Variables**	**Seropositive Rate**		
	**N**	**%**	***p *** value*
Gender			
Female (N = 76)	69	90.79	0.16
Male (N = 90)	75	83.33	
Age group			
< 7 years old (N = 22)	21	95.45	0.3
7–9 years old (N = 59)	52	88.14	
> 9 years old (N = 85)	71	83.53	
Urbanization level			
Suburban (N = 73)	60	82.19	0.13
Urban (N = 93)	84	90.32	
Parental occupation			
Skilled worker (N = 37)	28	75.68	0.03
Nonskilled worker (N = 109)	98	89.91	
Total (*N* = 166)	144	86.75	

*chi-squared test.

**Table 2:** **Risk-factor analysis of *Toxocara canis *** **infection among primary school children in the capital areas in the Republic of the Marshall Islands**

**Variables**	**Seropositive Rate**		
	**N**	**%**	***p *** **value**
Feeding dogs			
No (N = 7)	4	57.14	0.05*
Yes (N = 159)	140	88.05	
Cleaning dog huts			
No (N = 103)	88	85.44	0.52^#^
Yes (N = 63)	56	88.89	
Cleaning dog huts while wearing gloves			
No (N = 156)	136	87.18	0.62*
Yes (N = 10)	8	80.00	
Consuming frozen or raw food			
No (N = 15)	11	73.33	0.2*
Yes (N = 143)	125	87.41	
Touching soil			
No (N = 10)	7	70.00	0.13*
Yes (N = 155)	136	87.74	
Drinking untreated or unboiled water			
No (N = 32)	25	78.13	0.15*
Yes (N = 133)	118	88.72	
Consuming raw vegetables			
No (N = 30)	28	93.33	0.37*
Yes (N = 134)	114	85.07	
Total (*N* = 166)	144	86.75	

*Tested using Fisher’s exact test.

#chi-squared test.

**Table 3:** **Logistic regression analysis of *Toxocara canis *** **infection among primary school children in the capital areas of the Republic of the Marshall Islands**

	**Model I**			**Model II**		
**Variables**	**Crude ORs**^**a**^	**95% CIs**^**b**^	***p *** **value**	**Adjusted ORs**	**95% CIs**	***p *** **value**
Gender						
Male	1.00			1.00		
Female	1.97	0.76–5.12	0.16	2.57	0.86–7.65	0.10
Age groups						
> 9 years old	1.00			1.00		
7–9 years old	1.47	0.55–3.88	0.62	1.24	0.40–3.82	0.60
< 7 years old	4.14	0.51–33.36	0.24	3.26	0.38–28.23	0.30
Urbanization level						
Suburban	1.00			1.00		
Urban	2.02	0.81–5.04	0.13	2.70	0.87–8.28	0.08
Parental occupation						
Skilled worker	1.00			1.00		
Nonskilled worker	2.86	1.08–7.60	0.03	3.83	1.20–12.22	0.02
Feeding dogs						
No	1.00			1.00		
Yes	5.53	1.15–26.61	0.03	2.64	0.48–14.65	0.27

Model I: Univariate analysis, Model II: Multivariate analysis; ^a^Odd ratios; ^b^Confidence intervals.

Urbanization level was not significantly associated with seropositivity for *T. canis* in the uni- or multivariate logistic regression analyses (*p* > 0.05). By contrast, parental occupation level was a crucial contributing factor because PSC whose parents were employed as semiskilled workers exhibited a significantly higher risk of *T. canis* infection than did those whose parents were employed as skilled workers in both the univariate (ORs = 2.86, 95% CIs = 1.08–7.60, *p* = 0.03) and multivariate logistic regression analyses (ORs = 3.83, 95% CIs = 1.20–12.22, *p* = 0.02). Among the analyzed risk factors, univariate regression indicated that only PSC who exhibited a history of feeding dogs demonstrated an increased risk of *T. canis* infection compared with those who lacked such a history (ORs = 5.53, 95% CIs = 1.15–26.61, *p* = 0.03); by contrast, these effects were nonsignificant in the multivariate logistic regression analysis (ORs = 2.64, 95% CIs = 0.48–14.65, *p* = 0.27).

## Discussion

The RMI is a tropical developing country; thus, its climatic and living conditions might favor various pathogens survival including parasites such as *T. canis*. However, few systemic studies have evaluated the prevalence of *T. canis* infection among PSC in the RMI.

PSC are particularly vulnerable to toxocariasis because of their habits of playing in water or soil; eating raw foods; or contacting pets, including cats and dogs; thus, PSC are an ideal target group for investigating the prevalence of toxocariasis. Data collected from the evaluated age groups can be used to assess whether toxocariasis threatens the health of school-aged children, and can serve as a reference when evaluating the need for community interventions [23].

In most laboratories, such as the Centers for Disease Control and Prevention (CDC) in Atlanta, the current methods of detecting *T. canis* infection in humans are typically based on TcES-ELISA [6,24]. Although TcES-ELISA has been reported to yield reasonable levels of sensitivity (78%) and specificity (92%), when the threshold titer of positivity is set at 1:32 [18,19], the specificity is excessively low to be reliable when assaying communities in which several species of intestinal cross-reacting helminths (e.g., *Ascaris lumbricoides* ) are common. Although no attempt was made to verify whether the current participants were host to helminths other than *T. canis*, it was assumed that infection with several species of helminth would be common in the RMI. Thus, western blotting [19], rather than a general TcES-ELISA, was employed in this study. The findings indicated that *T. canis* infection was common among the PSC living in the capital areas of the RMI, and most PCS (86.75%) tested seropositive. Making a valid comparison of the present seroprevalence data with those recorded in previous studies is hampered by the variation in detection methods (ELISA or WB) and cut-off titers employed and by the general difficulty in exploring the relationships among titers, infection, and clinical disease [3,5,19]. Serological surveys conducted primarily among children in developed countries have indicated *T. canis* seroprevalence levels of 0.7% in New Zealand, 1.6% in Japan, 2.4% in Denmark, 7.5% in Australia, 14% in the United States, and 15% in Poland [3,5,25]. By contrast, high levels of seroprevalence have been reported in less developed, or tropical countries in Africa (30% in Nigeria, 45% in Swaziland, and 93% in La Reunion), Asia (81% in Nepal, 63.2% in Indonesia, and 58% in Malaysia), and South America (36% in Brazil and 37% in Peru) [21,26,27]. Other studies involving TcES-ELISA assessments have reported markedly increased seroprevalence levels of 77% among indigenous children in Taiwan [22] and 86% among children in St. Lucia [28]. In the present study, a relatively high cut-off titer (1:64) was used and it seems likely that some and perhaps many of the seropositive PSC exhibited active *T. canis* infections when they were sampled. According to the CDC, when conducting TcES-ELISA, a titer of 1:32 is indicative of active *Toxocara* infection [19].

Although boys might be more likely to be infected than girls in certain communities in which boys have frequent contact with dogs [15,26], the current results indicated that girls exhibited an elevated risk of infection. However, gender did not significantly affect seroprevalence in the present study, contrasting the results of studies in China, Iran, Nigeria, and Spain [16,29-31]. The reasons for this discrepancy warrant further investigation. Girls might often be involved in housework-related chores, such as washing clothes in water, or might have more frequent contact with dogs than boys do, increasing their opportunity to be exposed to *T. canis*; thus, subsequent studies are required.

The seroprevalence of *T. canis* infection in RMI PSC decreased as age increased; however, this trend was nonsignificant, possibly because of the small numbers of children in certain age groups. Seroprevalence might be cumulative in children because detectable titers of anti-*Toxocara* antibodies might persist over time postinfection [15,19,21]. No age-related increase in seroprevalence was observed among children in Argentina, Iran, Nigeria, or Spain [16,30,31].

Exposure to *Toxocara* is common because soil contamination is prevalent in peridomestic environments, and is exacerbated by poverty, poor hygiene, and the risk of contact with infected dogs [5,6]. The results of the full regression analysis suggested that poverty is a critical risk factor because seropositivity levels were elevated among PSC whose parents were employed as nonskilled workers (e.g., factory workers). A recent study verified that *Toxocara* infection is closely related to poverty status in the United States, suggesting that those in certain occupations, such as farming, might be at an increased risk [32].

A crucial risk factor of *T. canis* infection is contact with dogs [3]. The results of the univariate analysis in the current study indicated that PSC who exhibited a history of feeding dogs demonstrated a higher risk of *T. canis* infection than those who did not; however, these effects were nonsignificant in the multivariate regression analysis. Substantial evidence has indicated that direct human-dog contact might be a route of human infection based on the retained infectivity and pathogenicity of embryonated *T. canis* eggs recovered from dog hair [33-35]. Nagy et al. [36] located embryonated eggs on dog hair, but suggested that this route of transmission is rare. Nevertheless, a recent study indicated that embryonation is slow on dog hair but can occur; thus, transmission through direct contact, and even contact with well-groomed dogs, should not be ruled out [37]. Therefore, potential transmission through contact with embryonated *T. canis* egg contaminated in the environment such as soil should be not ignored also [3].

Children who have tested seropositive for *T. canis* have been linked to impaired cognitive development [25,38], generating cause for concern. Walsh and Haseeb [39] reported that seropositive children in the United States attained statistically lower cognitive scores on both the Wechsler Intelligence Scale for Children-Revised and Wide Range Achievement Test-Revised than did seronegative children. This finding was independent of socioeconomic status, ethnicity, gender, rural residence, cytomegalovirus infection, and lead levels in blood. Growing evidence has also implied that *T. canis* infection is associated with epilepsy [13,14]. Whether neurological deficits exist among PSC infected by *T. canis* in RMI warrants further comprehensive investigation.

## Conclusions

In summation, to prevent *T. canis* infections, PSC in the RMI must be educated regarding appropriate hygiene, including hand washing after contact with dogs and soil.

## Competing interests

The authors declare that they have no competing interests.

## Authors’ contributions

CKF, CJF, TWC, HSL, CHW, YCL, and MKL participated in the conception and design of the study; MYL and WWH participated in the analysis and interpretation of data; CKF and TWC drafted and revised the article; and CKF gave final approval of the version to be published. All authors read and approved the final version of the manuscript.

## Pre-publication history

The pre-publication history for this paper can be accessed here:

http://www.biomedcentral.com/1471-2334/14/261/prepub
